# Choriocarcinoma syndrome after resection of primary pulmonary choriocarcinoma: report of a case

**DOI:** 10.1186/s40792-016-0227-5

**Published:** 2016-11-02

**Authors:** Tsuyoshi Takahashi, Ryo Kobayashi

**Affiliations:** 1 Department of Surgery, Yaizu City Hospital, 1000 Dobara, Yaizu City, Shizuoka 425-8505 Japan; 2 Present address: Department of Thoracic Surgery, Graduate School of Medicine, University of Tokyo, 7-3-1 Hongo, Bunkyo-ku, Tokyo, 113-8655 Japan

**Keywords:** Lung cancer, Choriocarcinoma, Choriocarcinoma syndrome, Lung cancer surgery

## Abstract

**Background:**

Choriocarcinoma syndrome is known as a lethal complication from tumoral hemorrhage, which frequently occurs at the site of tumor metastasis.

**Case presentation:**

A 59-year-old man with 60-pack-year smoking history was referred to our hospital because of hemoptysis. Chest computed tomography (CT) showed a 28 × 18 mm spiculated nodule with a cavity infiltrating the left upper lobe. A transbronchial lung biopsy was performed, and histopathological examinations revealed adenocarcinoma. No distant or regional metastasis was observed, and therefore, the patient underwent a left upper lobectomy with lymphadenectomy. Histological examinations showed that the tumor consisted of poorly differentiated adenocarcinoma cells and a choriocarcinomatous component; no multiple pulmonary metastases and mediastinal lymph node metastasis were observed. Immunohistochemical analysis showed a positive immunoreaction for human chorionic gonadotropin in the syncytiotrophoblastic cells of the choriocarcinoma. One month after the operation, the patient developed massive hemoptysis. CT showed diffuse alveolar infiltration in both the lungs. A bronchoscopic examination showed bleeding from the right upper bronchus. Aspiration cytology showed carcinoma. Despite blood transfusion and management in the intensive care unit, the patient died one and a half month after diagnosis.

**Conclusions:**

We herein report a case of a man who developed choriocarcinoma syndrome 1 month after resection for combined choriocarcinoma and adenocarcinoma of the lung. Choriocarcinoma syndrome is a rare and life-threatening complication which may occur in patients with primary pulmonary choriocarcinoma. However, we need to consider the risk of this syndrome while dealing with patients who have massive hemoptysis.

## Background

Choriocarcinoma originates from anaplastic trophoblastic tissue and principally occurs in the female genital tract after a gestational event. Extragonadal choriocarcinoma is a rare entity; especially, primary pulmonary choriocarcinoma is an extremely rare entity. The lung is the most frequent site of metastasis from choriocarcinoma, and a lethal hemorrhagic comolication at the sites of metastasis is well-recognized. This is named the choriocarcinoma syndrome, containing high-volume choriocarcinomatous elements with siginificant elevation of serum levels of beta-human chorionic gonadotropin (hCG) [1].

## Case presentation

**Fig. 1:**
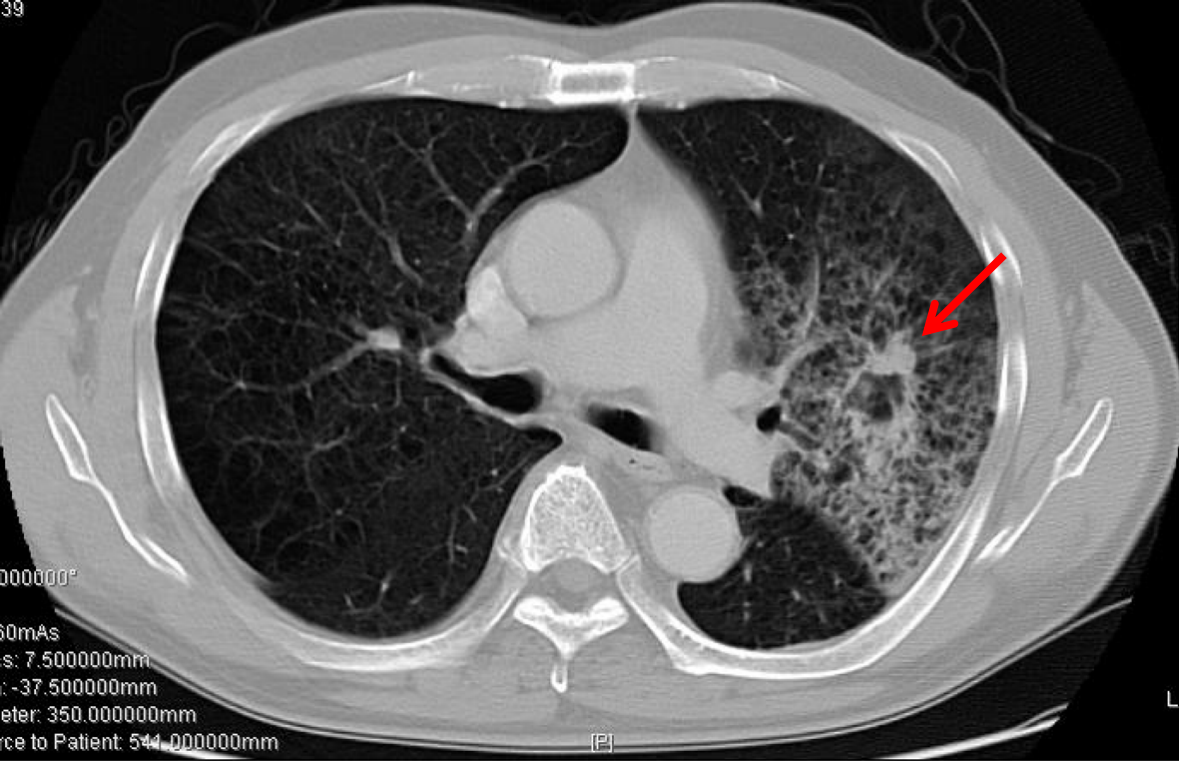
Chest computed tomography (CT) revealed a 28 × 18 mm spiculated cavitary nodule accompanied by parenchymal infiltration in the left upper lobe

**Fig. 2:**
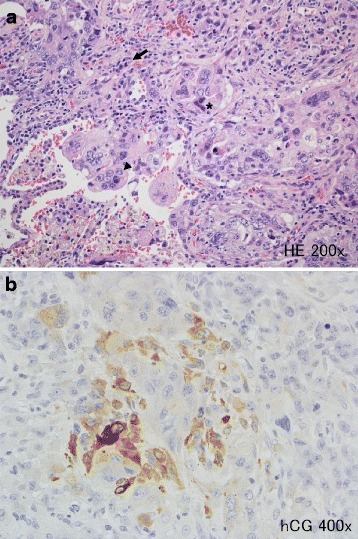
**a** Histologic findings showing that the tumor was consisted of the poorly diffenrentiated adenocarcinoma (*arrow*) and a choriocarcinomatous component comprised of cytotrophoblastic (*arrowhead*) and syncytiotriphoblastic cells (*) (hematoxylin and eosin stain; original magnification, ×200). **b** Immunohistochemistry showing a positive immunoreaction for beta-human chorionic gonadotropin (original magnification, ×400)

A 59-year-old man with a 60-pack-year smoking history was referred to our hospital due to hemoptysis. The patient had transurethral resection of the bladder tumor for bladder cancer. There were no abnormalities on physical examination, and laboratory data were within normal limits. His preoperative serum and urinary hCG levels were not examined. Chest computed tomography (CT) revealed a 28 × 18 mm speculated cavitary nodule accompanied by parenchymal infiltration (Fig. 1). Positron emission tomography (PET)/CT showed a fluodeoxyglucose uptake in the nodule. Transbronchial lung biopsy revealed adenocarcinoma of the lung. In the absence of distant and regional metastasis, the patient was diagnosed as having stage IA [cT1bN0M0] lung adenocarcinoma and underwent a left upper lobectomy with lymphadenectomy. Histologic findings showed that the tumor was consisted of the choriocarcinomatous component (cytotrophoblastic and syncytiotriphoblastic cells) and poorly diffenrentiated adenocarcinoma (Fig. 2a). There were no multiple pulmonary metastasis and mediastinal lymph node metastasis. Immunohistochemistry showed a positive immunoreaction for hCG in the syncytiotrophoblastic cells of the choriocarcinoma (Fig. 2b).

**Fig. 3:**
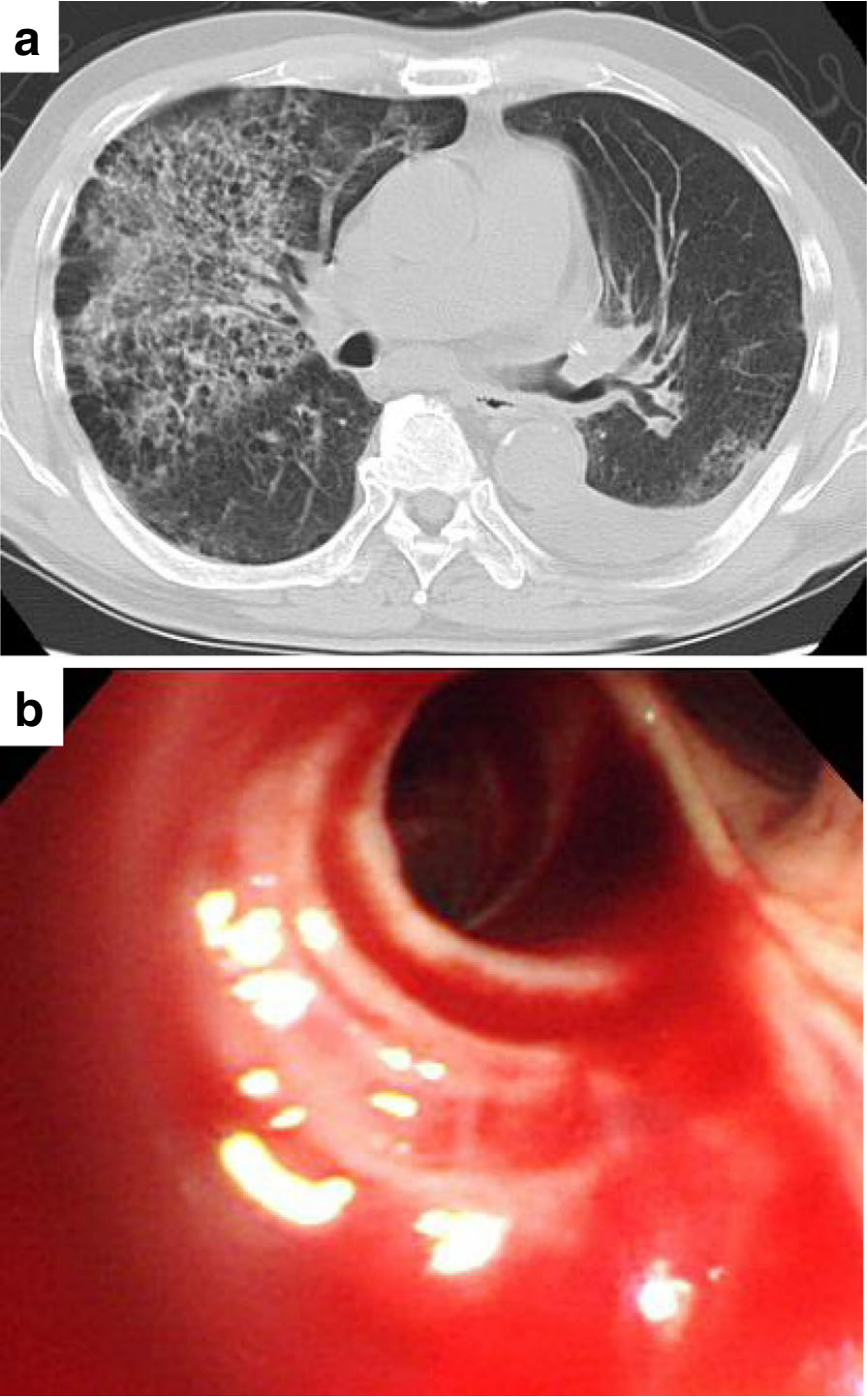
**a** Chest computed tomography (CT) revealed diffuse alveolar infiltrates in the both lungs. **b** Bronchoscopic examination revealed massive bleeding from the right upper bronchus

The patient’s postoperative course was uneventful. One month after the operation, the patient developed massive hemoptysis. CT revealed diffuse alveolar infiltrates in the both lungs (Fig. 3a). Bronchoscopic examination revealed massive bleeding from the right upper bronchus (Fig. 3b). Aspiration cytology from the right upper bronchus showed a choriocarcinoma component. Despite blood transfusion and management in the intensive care unit, the patient died.

### Discussion

Choriocarcinoma is a germ cell tumor containing syncytiotrophoblastic cells and secreting hCG and most commonly occurs in the female genital tract after a gestational event such as hydatidiform mole, spontaneous abortion, ectopic pregnancy, or term pregnancy [2]. Primary pulmonary choriocarcinoma is an extremely rare entity and the pathogenesis of primary pulmonary choriocarcinoma has not been established yet. In our case, the tumor was consisted of a choriocarcinomatous component (cytotrophoblastic and syncytiotriphoblastic cells) and poorly diffenrentiated adenocarcinoma. To our knowledge, only one case of combined choriocarcinoma and adenocarcinoma of the lung has been reported by Chen et al. so far [3]. Chen et al. reported that there is the possibility that choriocarcinoma and adenocarcinoma arouse simultaneously or formed a collision tumor; however, it is considered that the choriocarcinoma might occur directly in the lung through trophoblastic differentiation of some cells in the former adenocarcinoma.

No established therapeutic guidelines are available for this clinical entity due to its rarity. In a small pulmonary choriocarcinoma, the first choice in the treatment may be surgical resection [4]. However, the natural course of choriocarcinoma is rapidly fatal in the great majority of cases, especially some patients presenting with choriocarcinoma syndrome which is characterized by life threatening bleeding at the sites of metastasis from choriocarcinoma may be threatened immediately by the complications of pulmonary hemorrhage. Hemoptysis, elevation of serum levels of hCG and the roentgenographic infiltrates with typical metastatic sharply defined nodules are characteristic with the choriocarcinoma syndrome [1]. In our case, unfortunately elevation of serum levels of hCG could not be confirmed because the patient suffered from massive hemoptysis and died before the pathological diagnosis of combined choriocarinoma and adenocarcinoma of the lung was made. Though chest CT revealed no pulmonary nodules suggestive of metastasis, we could make a definite diagnosis of choriocarcinoma syndrome from aspiration cytology. There is a report that combined modality therapy consisting of chemotherapy including bleomycin, etoposide, and cisplatin along with surgery and irradiation may result in a complete response [5]. Early recognition of choriocarcinoma syndrome and the urgent multimodality treatment consisted of surgery and chemotherapy might be beneficial.

## Conclusions

Choriocarcinoma syndrome is a rare and life-threatening complication which may occur in patients with primary pulmonary choriocarcinoma. However, we need to consider the risk of this syndrome while dealing with patients who have massive hemoptysis.

## Consent

Written informed consent was obtained from the patient family for publication of this case report and any accompanying images. A copy of the written consent is available for review by the Editor-in-Chief of this journal.

## Abbreviations

CT: Computed tomography
hCG: Human chorionic gonadotropin
PET: Positron emission tomography
